# Development of an advance directive ’communication tool’ relevant for patients with advanced cancer in six European countries: Experiences from the ACTION trial

**DOI:** 10.1371/journal.pone.0271919

**Published:** 2022-07-28

**Authors:** Caroline Moeller Arnfeldt, Mogens Groenvold, Anna Thit Johnsen, Branka Červ, Luc Deliens, Lesley Dunleavy, Agnes van der Heide, Marijke C. Kars, Urška Lunder, Guido Miccinesi, Kristian Pollock, Judith A. C. Rietjens, Jane Seymour, On behalf of the ACTION consortium

**Affiliations:** 1 Department of Public Health, University of Copenhagen, Copenhagen, Denmark; 2 Palliative Care Research Unit, Department of Geriatrics and Palliative Medicine (GP), Bispebjerg and Frederiksberg Hospital, University of Copenhagen, Copenhagen, Denmark; 3 Department of Psychology, University of Southern Denmark, Odense, Denmark; 4 University Clinic for Respiratory and Allergic Diseases Golnik, Golnik, Slovenia; 5 End-of-Life Care Research Group, Vrije Universiteit Brussel & Ghent University, Ixelles, Belgium; 6 International Observatory on End of Life Care, Lancaster University, Lancaster, United Kingdom; 7 Department of Public Health, Erasmus MC, Rotterdam, The Netherlands; 8 Julius Center for Health Sciences and Primary Care, University Medical Center Utrecht, Utrecht University, Utrecht, The Netherlands; 9 Center for Oncological Network, Study and Prevention of Cancer (ISPRO), Florence, Italy; 10 School of Health Sciences, University of Nottingham, Nottingham, United Kingdom; 11 Division of Nursing and Midwifery, Health Sciences School, University of Sheffield, Sheffield, United Kingdom; Murcia University, Spain, SPAIN

## Abstract

**Background:**

The ACTION trial evaluated the effect of a modified version of the Respecting Choices´ advance care planning programme in patients with advanced cancer in six European countries. For this purpose, an advance directive acceptable for all six ACTION countries to be used for documenting the wishes and preferences of patients and as a communication tool between patients, their caregivers and healthcare staff, was needed.

**Aim:**

To describe the development of a multinational cancer specific advance directive, the ´My Preferences form´, which was first based on the 2005 Wisconsin ‘Physician Orders of Life Sustaining Treatment´ Form, to be used within the ACTION trial.

**Methods:**

Framework analysis of all textual data produced by members of the international project team during the development of the ACTION advance directives (e.g. drafts, emails, meeting minutes…).

**Setting/participants:**

ACTION consortium members (N = 28) with input from clinicians from participating hospitals (N = 13) and ´facilitators´ (N = 8) who were going to deliver the intervention.

**Results:**

Ten versions of the ACTION advance directive, the ´My Preferences form´, were developed and circulated within the ACTION consortium. Extensive modifications took place; removal, addition, modification of themes and modification of clinical to lay terminology. The result was a thematically comprehensive advance directive to be used as a communication tool across the six European countries within the ACTION trial.

**Conclusion:**

This article shows the complex task of developing an advance directive suitable for cancer patients from six European countries; a process which required the resolution of several cross cultural differences in law, ethics, philosophy and practice. Our hope is that this paper can contribute to a deeper conceptual understanding of advance directives, their role in supporting decision making among patients approaching the end of life and be an inspiration to others wishing to develop a disease-specific advance directive or a standardised multinational advance directive.

## Background

The concept of advance care planning (ACP) has gained variable currency in policy and practice internationally. Rietjens et al. provided the following definition of ACP: ‘*Advance care planning enables individuals to define goals and preferences for future medical treatment and care*, *to discuss these preferences with family and health-care providers*, *and to record and review these preferences if appropriate*’ [1]. One way of recording such preferences is with an advance directive (AD). An AD is an umbrella term for documents completed by healthy or ill persons with legal capacity which contain provisions for medical treatment and care in the future event that the person loses the capacity to make his or her own decisions [2]. Availability, medical relevance, clarity and comprehensiveness are considered important properties of ADs [3]. ADs are bound by national clinical practices and associated legalities and are therefore known to be country or state specific. The majority of ADs are generic across diseases, but some are also found to be disease specific [3–6].

In the ACTION trial, assessing the effect of (a modified version of) the Respecting Choices (RC) ACP programme across six European countries (Belgium, Denmark, Italy, the Netherlands, Slovenia and the United Kingdom), an AD acceptable to all partners was needed. However, considerable differences both in relation to levels of knowledge of, experience with and implementation of ACP and ADs across the six ACTION countries existed; in Denmark and the Netherlands, the first version of an AD was implemented in 1992 [7] and 1995 [8], respectively. In Denmark, the knowledge and use of the national Living Will had nonetheless remained low and the overall acceptance mixed [9]. In Belgium, Slovenia and the United Kingdom, ADs were regulated by law or formalised via statue respectively in 2002 [10], 2008 [11] and 2005 [12]. In Italy, ADs were first regulated by law in 2017 [13]. In addition, while systematic ACP was a relatively familiar concept within for example the United Kingdom, the concept of structured and formalized ACP conversations was largely unknown in Denmark and Slovenia at the start of the ACTION trial in 2013 [14, 15].

In this article, the development of a joint and standardized AD to be used as a documentation and communication tool for patients with advanced cancer as part of the ACTION trial will be described. The purpose of the ACTION AD was to have a standardised uniform document that allowed for documentation of the wishes and preferences expressed by patients during the ACTION Respecting Choices´ advance care planning conversations and could be used as a communication tool between patients, their caregivers and healthcare professionals. The AD had to be acceptable for all ACTION partners, remain standardised across the six countries and be transparently connected to the ACTION RC ACP intervention (and its related documents). In Table 1, an overview of the topics presented within the ACTION Respecting Choices advance care planning conversations, as well as sample questions, are presented. As no such AD existed, a form was developed. Accounts and detailed information about the development or adaptation of ADs are sparse. With few exceptions [5, 6, 16], the descriptions available are short. The aim of this article is to provide a detailed description of how the multinational cancer specific ACTION AD, the ´My Preferences form´ (MPF) was developed as well as presenting the substantive discussions and insights coming from developing this communication tool.

**Table 1:** Topics in the ACTION Respecting Choices ACP conversations.

Topic	Sample question
1. Understanding of role of the PR	What do you understand about the role of the Personal representative?
2. Patient’s and PR’s understanding of ACP	Have you done any Advance Care Planning before?
3. Understanding of illness	Tell me what you understand about your illness
4. Complications	What do you understand about the possible complications of your illness and what might happen in the future?
5. Experiences	What did you learn from that experience [experiences with family or friends who became ill or injured and were not able to communicate]?
6. ‘Living well’	What does living well mean to you?
7. Worries and fears	Do you have worries about your illness or medical care? If so, what worries do you have?
8. Possible personal, cultural, religious, or spiritual beliefs	Do you have any personal or cultural beliefs that might influence your preferences for future care and treatment?
9. Patient’s hopes for current medical plan of care (part 1)	What do you hope for with your current medical plan of care?
10. Patient’s hopes for current medical plan of care (part 2)	I understand these hopes. If all these hopes do not come true, what else would you hope for?
11. Help making an informed decision regarding CPR	What do you understand about resuscitation?
12. Discuss goals, values and preferences for future complications	Tell me in your own words what you understand about this option [Selective Treatment plus Comfort-Focused Care]?
13. Preferences relating to final place of care	Do you have preferences relating to the final place of your care?

Reprinted with permission from Zwakman et al. [17] and under the Creative Commons Attribution 4.0 International License (http://creativecommons.org/licenses/by/4.0/)

## Methods

### Context of development

The My Preference Form (MPF) was developed within and to be used in the ACTION trial. The ACTION trial was a phase III multicentre cluster randomised clinical trial that evaluated the effect of a modified version of the Respecting Choices (RC) advance care planning (ACP) intervention in six European countries: the Netherlands, Belgium, Italy, Slovenia, United Kingdom and Denmark. The RC ACP programme from La Crosse, Wisconsin, in United States is recognized as a comprehensive and structured ACP programme (https://respectingchoices.org/). Within the ACTION trial, an adapted version of the RC ACP programme, combining the RC First Steps and Advanced Steps were evaluated. In total, 23 hospitals, twelve intervention hospitals and 11 control hospitals included patients from 2015 to 2018. The ACTION patient population consisted of competent adult patients with advanced lung (small cell or non-small cell, stage III and IV) or colorectal cancer (stage IV or metachronous metastases) with a WHO performance status of 0–3 and an anticipated life expectancy of >3 months. Patients received the ACP intervention as one or more structured conversations with a trained facilitator. Based on patient preferences, a caregiver of the patient, a ‘personal representative’ (PR), was also invited to participate. During the conversations, the MPF was introduced to the patient and his or her PR. Additional information about the ACTION trial (Trial Number: ISRCTN63110516) and the ACTION RC ACP intervention is presented in S1 File and in a protocol article [18].

The first discussions about the ACTION AD, the MPF, took place between country representatives (consortium members and clinicians from the different ACTION countries) during RC ACP intervention training sessions held in Wisconsin, the United States, in May 2014. The first version of the MPF was circulated within the ACTION consortium in August 2014. It focused on four themes: cardiopulmonary resuscitation, life-prolonging treatment, artificial nutrition and fluids and information about whom the form had been discussed with (S2 File: My Preferences Form (version 1)).

From the start of the ACTION trial in December 2013 to March 2015 when the MPF was finalized, 10 versions were circulated within the ACTION consortium. During this period of time, especially two key sources were drawn upon: the 2005 Wisconsin ‘Physician Orders of Life Sustaining Treatment Form’ (POLST) [19] and the ´La Crosse Region Power of Attorney for Healthcare Document´ (POA-HC) [20]. The reason that these two documents were applied as the primary inspiration sources was that they had been used as part of the Respecting Choices training in La Cross, Wisconsin, US. The POLST is a medical orders form for patients who are serious ill, frail or at the end of life, completed by health care professionals together with the patient, whereas the POA-HC is an AD, which can be completed by all competent adults. The POLST form was the major inspiration source applied for version 1, whereas the POA-HC was used as a basis for version 3.

To accommodate the different local regulations in relation to ACP and ADs in the six ACTION countries, it was decided that the MPF could either be applied as a legal AD, as an addition to local forms or simply used as a communication tool. As such, we also refer to the MPF as an AD ’communication tool’ several places throughout this article. Completion of the form was optional. The form was to be patient-held, but could be transferred to the medical file and shared with health care professionals on the patient´s initiative.

Information about how patients completed the MPF and comparison of their goals and preferences across countries is provided by Zwakman et al. [21].

### Data sources

This paper is fully data driven, based on analysis of all written ACTION documents and materials officially shared among consortium members relating to the development of the MPF. Three groups of people participated in written feedback and discussions and can thus be considered the “data sources”: the ACTION consortium members (the authors of this article included), clinicians from the hospitals participating in the trial and the facilitators from each of the countries who delivered the intervention. As clinicians and facilitators provided written feedback on one occasion only during the development phase of the MPF, the ACTION consortium members can be considered the main data source. For characteristics of the three groups, see Table 2. As this article is based upon written internal communication within the ACTION consortium not originally planned to be used as data, consent has been obtained with the constraint that quotes are only linked to one of the three groups of people providing the data material.

**Table 2:** Overview of the groups of people who contributed to the development of the My Preferences Form (MPF).

Groups	Short description of people within the groups
**The ACTION consortium**	The ACTION consortium consisted of 28 researchers (professors, senior researchers and junior researchers) and clinicians from multidisciplinary backgrounds within medicine, nursing, public health and social science from Belgium, Denmark, Italy, the Netherlands, Slovenia and United Kingdom
**Clinicians from hospitals**	13 physicians and nurses (primarily physicians) specialized in either oncology or palliative care from Belgium, Denmark, Italy, the Netherlands, Slovenia and United Kingdom
Facilitators	Eight female Dutch facilitators, who were all nurses and primarily specialised in oncology.

### Data collection and data material

In order to collect all written feedback and discussions relating to the development of the MPF, 1,164 emails and their attachments circulated within the ACTION consortium from December 2013 to March 2015, where the final version was circulated, were scrutinized. From this process, 76 documents with content about or related to the development of the MPF were identified and extracted: 10 versions of the MPF and 66 documents with overall information about the development of the MPF (Table 3).

**Table 3:** Overview of the various kinds of textual data that was applied and coded.

Type of document	Number of documents (N = 76)
	
The official versions of the MPF	10
	
E-mails	17
Consortium meeting minutes (both revised and final)	10
MPF with comments/corrections	11
ACTION RC ACP conversation guides with comments/corrections	14
Original RC material	2
Teachers´ meeting minutes	3
Summaries about planning and decisions made	2
Feedback proposals in relation to the MPF/the conversation guides or the intervention in general	6
Summary of pre-test with clinicians in all countries	1

### Ethical considerations

Ethical approval for the ACTION trial was obtained from the institutional review board (IRB) of the coordinating centre (‘Medische Ethische Toetsings Commissie (METC) Erasmus MC’) and the IRBs of the five ACTION countries. In addition, all ACTION consortium members provided written informed consent through email allowing for their internal communication (email, meeting minutes etc.) to be used as data in the article.

### Data analysis

The data analysis consisted of two steps: first a systematic comparison of the 10 versions of the MPF, followed by a qualitative analysis of the remaining 66 documents.

#### The descriptive level—the systematic comparison

**Fig 1:**
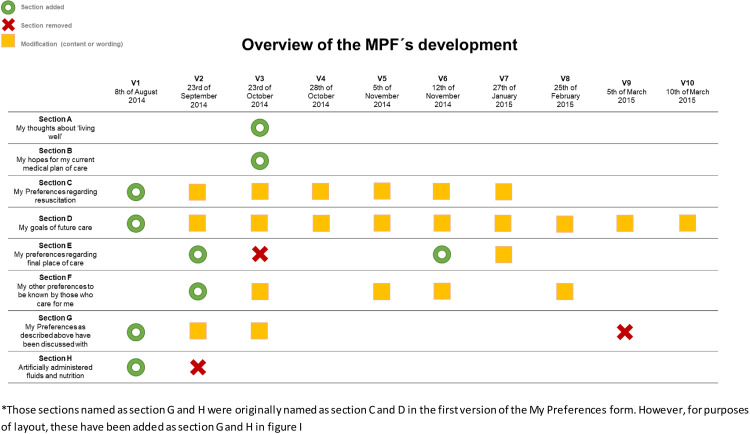
Overview of the thematic development of the My Preferences Form (MPF).

The systematic comparison of the 10 versions of the MPF was carried out in Excel, listing the versions side by side, allowing for a clear visualization of the development in between versions and of the overall development. From this overview, a figure summing up the development (presented as Fig 1 in the Results section) and a written comprehensive descriptive summary of the development and modifications of each section was made.

#### The qualitative analysis—framework analysis

The framework approach by Richie and Lewis [22–24] was used to analyse the textual data from the development process. The approach has five steps; familiarization, identifying a thematic framework, indexing, charting, mapping and interpretation [22–24]. The approach is considered flexible, yet systematic and is known for its ability to reduce large amounts of various kinds of data through thematic frameworks and matrices while remaining transparent and grounded in data [23].

The first author (CMA) carried out all steps of the analysis, while two experienced and senior researchers with qualitative research experience (JS and ATJ) participated in selected steps and provided continuous supervision. The framework approach was used flexibly as it was adjusted to the focus of the research.

The first step, the familiarization process, was linked to the descriptive level of the analysis and built on immersion in and examination of all the data, notetaking and a systematic comparison of all the versions of the MPF. Based on this step and discussions between the CMA and R1 and R2, an initial thematic framework was made in step two. The initial thematic framework consisted of 19 codes, some of which were descriptive while others were analytical. This initial thematic framework was tested by CMA and R2, discussed by CMA, R1 and R2, and subsequently refined. This alteration of the thematic framework meant that additional codes, emerging from the testing itself, were added, and that the more analytical codes were deleted, as they did not work well in practice at this stage of the analysis. In step three, the final thematic framework, now consisting of 20 codes (Table 4), was applied to the 66 documents by CMA using the qualitative analysis software programme NVivo 11. In step four, CMA transferred all coded data into seven theme-based matrices, either as summaries of data or “raw” data, linking theme (the codes) and case (versions of the MPF). This provided an overview of the discussions taking place at the different steps of the development process. In step five, matrixes were reviewed and interpretation notes were made.

**Table 4:** The final coding framework.

**Name of main codes and sub codes**	**Description of main codes and sub codes**
Adaptation	Data where the need for adaptation is mentioned or discussed
Aim (of My Preferences form)	Data about what kind of document we need and strive for
Health care agent	Data about the Health Care Agent
Sharing and adapting the form	Data about how to adapt the form, how to share the form and who is responsible for the form.
Local ACP—or the lack of it	Data about official ACP practices within the ACTION countries—or the lack of it
Structure and design of the form	Data about how the form should be structured and designed (open questions, ticking boxes etc.)
Other	Data which do not fit within one of the current nodes
Relevant data from the conv. guides	Data from the RC ACP conversation guides that are relevant for the development of the intervention or the MPF
Section A (living well)	Data about the inspiration of section A, its content and its use
Indirectly about section A	Data indirectly about section A´s development, use and purpose
Section B (hopes)	Data about the inspiration of section B, its content and its use
Indirectly about section B	Data indirectly about section B´s development, use and purpose
Section C (Cardiopulmonary resuscitation)	Data about the inspiration of section C, its content and its use
Section D (goals of care)	Data about the inspiration of section D, its content and its use
Section E (Final place of care)	Data about the inspiration of section E, its content and its use
Section F (other preferences)	Data about the inspiration of section F, its content and its use
Section G (discussed with)	Data about the inspiration of section G, its content and its use
Section H (Artificial nutrition and hydration)	Data about the inspiration of section H, its content and its use
Views about ACP and ADs	Data about views, statements and discussions about ACP and ADs which directly or indirectly shows how people understand ACP and ADs
When to use to form (capacity issue)	Data about when the form should be applied

## Results

### The final version of the My Preferences form

The final version of the MPF (version 10) was circulated in March 2015. It was a comprehensive form that aligned with the ACTION RC ACP conversation guides to be applied by facilitators during the ACP conversations. The final version of the MPF form contained six themes: the patient´s thoughts about living well, hopes for the current medical plan of care, cardiopulmonary resuscitation, life prolonging-treatment, preferences for final place of care and other general preferences (S3 File: My Preferences Form (version 10)).

From the first to the final version of the MPF only two (highly revised) themes remained: cardiopulmonary resuscitation and life-prolonging treatment. Fig 1 gives an overview of the development of the themes (sections) included in versions 1–10 of the MPF. This figure shows that four sections were added and three sections were removed (one only to be reintroduced) during the development process. The sections added were: the patient´s thoughts about living well, hopes for the current medical plan of care, preferences for final place of care and other general preferences. The sections removed were: artificially administered fluids and nutrition, ‘my preferences as above have been discussed with’, and final place of care (the latter reintroduced). Furthermore, in 22 cases, at least one or more modifications were made in relation to either content or wording within a section.

Below the development of the individual sections within the MPF is described in detail.

#### Section A my thoughts about living well and section B my hopes for my current medical plan of care

What ended up as section A ´My thoughts about living well´ and section B ´My hopes for my current medical plan of care´ was added to version 3 of the MPF. The sections were added based on discussions about how to adapt the RC intervention materials and the MPF to the needs of patients with advanced cancer. As argued by a consortium member: ‘*In our view*, *the proposed ACTION document [version 1] misses most of the important issues when it concentrates on CPR and end of life care only’*. As a result, a focus on not only future, but also the current situation and treatment preferences was incorporated into the MPF with the topics of ‘living well’ and the patient´s ´hopes for the current medical plan of care´. The ‘living well’ topic was taken from the ACTION RC ACP conversation guide. It contained three questions for the patient to answer: 1) activities or experiences that were important to living well, 2) fears or worries and 3) cultural, religious or spiritual beliefs. The ‘hope’ topic was imported from the RC ACP ´Next Steps´ curriculum, which was not originally meant to be applied within the ACTION trial, but was added with recommendation and permission from the RC organisation. This expansion of topics within the MPF and the ACTION RC conversation guides was not done without hesitation as expressed by a consortium member: *‘[…] we need to be very careful not to turn the conversation into a general conversation about palliative care needs and concerns’*. The consortium member thereby highlighted the risk of taking away focus from what should happen if the patient lost his or her capacity.

In summary, it was decided to add sections A and B to make the MPF as relevant as possible to the needs and situation of patients with advanced cancer.

#### Section C my preferences regarding cardiopulmonary resuscitation (CPR)

Section C was extensively discussed. The first major discussion arose after the first version was circulated. It centred on whether cardiopulmonary resuscitation (CPR) should be part of the MPF at all. As one consortium member argued *‘[…] CPR is less important for cancer patients*, *hence the strong focus on CPR in the document […] is somewhat less relevant’*. While questioning the clinical relevance of CPR in relation to the ACTION patient population, among whom the cause of death is likely to be advanced cancer, as well as considering the low chances of survival after CPR when having advanced cancer [25–27], the topic nonetheless remained part of all 10 versions of the MPF.

Throughout the 10 versions, section C was modified on six occasions (see Fig 1). Most of these modifications included changes to the number of options provided in relation to cardiopulmonary resuscitation and the wording of these (see Table 5, section C).

**Table 5:** Overview of the main modifications made within the My Preferences Form (MPF).

**Main modifications made to the sections that ended up as part of the final version of the MPF**					
**Section A**	**V3**				
My thoughts about living well	Activities or experiences that are important for me to live well:				
	I have the following fears or worries:				
	I have the following cultural, religious or spiritual beliefs:				
**Section B**	**V3**				
My hopes for my current medical plan of care	My hopes for my current medical plan of care include:				
**Introduction text to section C-F**	**V7**				
	If I were to become unable to communicate and express my preferences I would like the following issues to be taken into account (sections C-F)				
**Section C**	**V1**	**V2**	**V3**	**V4**	**V6**
My preferences regarding resuscitation	Treatment preferences when I am not breathing and have no pulse: Resuscitate. Do not attempt or continue any resuscitation.	Wishes and concerns regarding CPR:	Cardiopulmonary Resuscitation (CPR): • I have an incurable illness or injury and am dying; OR • I have no reasonable chance of survival if my heart stops; OR • I have little chance of long-term survival if my heart stops and the process of resuscitation would cause significant suffering. I do not want CPR attempted if my heart or breathing stops.	My preferences regarding resuscitation: I want to be resuscitated. I want CPR attempted unless my physician determines one of the following: I want CPR attempted unless my physician determines one of the following (*scenarios are identical to those described in V3*). I do not want CPR attempted if my heart or breathing stops.	My preferences regarding resuscitation: I wish to have CPR attempted if my physician considers it medically appropriate in my actual situation. I do not wish to have CPR attempted if my breathing or heart stops.
**Section D**	**V1**			**V2**	
My goals of future care	Treatment preferences when I have a pulse and/or am breathing Comfort Care only: I am treated with dignity, respect and kept clean, warm and dry. Food and fluids are offered by mouth, but not forced upon me. Attention is paid to hygiene. Medication, positioning, wound care, and other measures are used to relieve pain and suffering. Oxygen, suction and manual treatment of airway obstruction may be used as needed for comfort. These measures are to be used where I live. If comfort measures fail, contact my physician. Comfort Care with Limited Additional Interventions aimed at prolonging my life: Includes comfort care as described above. May include cardiac monitoring and oral/IV medications. Transfer to hospital if indicated, but no endotracheal intubation or long term life support measures. I understand that this type of care usually does not involve admission at an intensive care unit. Full Treatment to Prolong my Life: Includes comfort care as described above plus measures that may prolong my life, such as endotracheal intubation, advanced airway, and cardioversion/automatic defibrillation at the intensive care unit. Other instructions:			Wishes in relation to future treatment:	Wishes and concerns regarding life sustaining-treatment:
	**V3**		**V5**		**V6**
	Goals of future care When I have lost the capacity to make my own decisions and have a complication that I am unlikely to live through (survive), I prefer the following measures to be taken: Comfort Care only. Allow natural death. My care should include keeping me comfortable, clean, and warm. I prefer reasonable measures to offer me food and water that I can take by mouth. I would like medications, positioning, wound care, and other measures that relieve my pain and suffering. Oxygen, suction, and other simple treatments to treat anything blocking my ability to breathe may be used for purposes of comfort. I prefer these treatments to be provided where I live. I only want to be hospitalized if there is no other way to keep me comfortable. Comfort Care with Limited Additional Interventions. In addition to the comfort measures listed above, cardiac monitors, oral, and IV medications may be used to help my heart and breathing and I may be transferred to the hospital if needed. I would prefer not to be intubated and not to go to the intensive care unit. All Available Treatment to Restore my Heart, Lung, and Other Organ Systems. In addition to the comfort and other treatments listed above, I prefer the use of all measures including a tube into my lungs to help me breathe, a breathing machine, and all treatments available in an intensive care unit.		*The description scenario is identical to the one applied in V3*. Comfort Care. Allow natural death. *Only minor change in wording*: However, I may be hospitalized if there is no other way to keep me comfortable. Comfort Care with Additional Interventions. Broadly speaking this would mean that in addition to the comfort measures listed above, I agree with any additional intervention my physician determines that might improve my condition.	My Goals of future care In the case I have lost the capacity to make my own decisions and have a complication that I am unlikely to live through, I prefer the following measures to be taken: Comfort Care Comfort care. Allow natural death. Broadly speaking this would mean that my care should include keeping me comfortable, clean, and warm. I prefer reasonable measures to offer me food and water. I would like medications, positioning, wound care, and other measures that relieve my pain and suffering. Oxygen, suction, and other simple treatments to treat anything blocking my ability to breathe may be used for purposes of comfort. Comfort Care with Additional Interventions *Modification*: might prolong my life/might improve my condition	
	**V7**		**V8**	**V9**	**V10**
	My goals of future care I know that it happens to some patients that they experience a complication. Should I have such a complication while I am unable to communicate and express my preferences, the following option best fits my goals and values: Comfort Focused Care Primary goal of maximizing comfort (natural death may occur). The focus of this option is to relieve pain and suffering. This may include medications and other simple treatments to treat any symptoms I may have. I understand that this may involve transfer to hospital only if my comfort needs cannot be met in my current location. Selective Treatment Primary goal of attempting to treat the complication This includes all treatment to keep me comfortable as described in Comfort-Focused Care. This also includes other interventions the physician thinks might help me recover from the complication. I am aware that some of these treatments may require admission to hospital.		*The description scenario is identical to the one applied in V7*. Comfort Focused Care *Modification*: *deletion of ‘natural death’ and replacements of ‘treatments’ with ‘interventions’*. Selective Treatment *The text is identical to the one in V7.*	*Modification*: *‘Potentially life-threatening’ has been added to describe the complication in the description scenario*. Selective Treatment plus Comfort Focused Care. Primary Goal of attempting to treat the complication. I would like my physician to give it a try and provide me with interventions he thinks might help me recover from the complication and extend my life. In addition to that, pain and suffering will be relieved. This may include medications and other simple interventions to treat any symptoms I may have. I understand that any of these treatments may involve transfer to hospital. Comfort Focused Care only *Modification: ‘only’ has been added: ‘The only focus of this option is to relieve pain and suffering’*. (Clarify or elaborate below, if appropriate)	My goals of future care I know that it happens to some patients that they experience a potentially life-threatening complication. Should I have such a complication while I am unable to communicate and express my preferences, the following option best fits my goals and values: Selective Treatment plus Comfort Focused Care. Primary Goal of attempting to treat the complication I would like my physician to provide me with interventions he thinks might help me recover from the complication and extend my life. In addition to that, pain and suffering will be relieved. This may include medications and other simple interventions to treat any symptoms I may have. I understand that any of these treatments may involve transfer to hospital. Comfort Focused Care Primary goal of maximizing comfort. The focus of this option is to relieve pain and suffering. This may include medications and other simple interventions to treat any symptoms I may have. I understand that this may only involve transfer to hospital if my comfort needs cannot be met in my current location. (Clarify or elaborate below, if appropriate)
**Section E**	**V2**		**V6**	**V7**	
My Preferences regarding final place of care	Preferred place of care at the end of life:	Preferred place of death:	My preferences regarding place of care I have a preferred place of care. This place is: I do not have a preferred place of care.	My preferences regarding final place of care. I have a preferred final place of care. This place is: I do not have a preferred final place of care.	
**Section F**	**V2**		**V3**	**V5**	**V6**
My other preferences that I consider important to be known by those who care for me	Wishes and concerns in relation to end of life/death:	Whatever else is important for you to be known by those who care for you (no matter how trivial it is):	My other preferences that I consider important to be known by those who care for me: (*Instructions*: these may relate to e.g. place of care/place of death)	*Identical to text in V3*. *(Instructions*: may relate to e.g. place of care/place of death’ and ‘use of or refusal of any specific interventions)	*Identical to text in V3*. *(Instructions*: may relate to e.g. use of or refusal of any specific interventions)

Overall, several discussions took place highlighting implications and challenges associated with the overall concept of the form. As an example, it was discussed what format was preferred (ticking boxes or free text). Another lengthy and recurrent discussion was whether patients should be allowed to have all choices possible as seen in relation to, for example, the topic of CPR. In this relation two main arguments were brought forward: the first relying on the fact that patients across the six ACTION countries could not (and still can´t) demand CPR, since this is a medical decision taken by the treating physician, who has ´the obligation to deliver only appropriate treatment´ [28], albeit informed by the patient´s views and wishes. The second argument focused on the ethical obligation to offer CPR as a real choice as part of hearing our patients´ goals and preferences: *‘Section C of ‘My Preferences’ form*, *as it is now [version 3]*, *does not allow to freely choose CPR because it does not include the ‘free’ option ‘I want CPR attempted if my heart stops’ […]’*. Following this line of arguments, the option ‘I want to be resuscitated’ was added in version 4. However, the wording of this option was again criticized for potentially conflicting with the physicians´ ultimate decision-making capacity on cardiopulmonary resuscitation. As Illustrated in Table 5, this specific discussion went back and forth and several solutions were applied. In addition, the status of the form, which was originally meant to be patient-held, and therefore not routinely included in the hospital records, also led to discussion. As highlighted by a consortium member reporting clinician feedback: *‘The DNAR [do not attempt resuscitation] needs to be communicated with clinical teams immediately*. *This should not be the patient’s responsibility´*. Ultimately, section C ended up with two options in relation to cardiopulmonary resuscitation emphasising that the physician has the decision-making capacity (Table 5, section C, version 6). The illness and survival scenarios previously described in versions 3 and 4 were left out (Table 5, section C, version 3–4), as the interpretation of these scenarios differed among consortium members. In relation to the status of the form as a patient-held form, the solution ended up being pragmatic: most ACTION countries used the form as a patient held form which could be shared with medical staff (BE, IT, NL, SI, UK) while Denmark aimed to transfer the goals and preferences expressed directly into the medical files with consent of the patient.

In summary, section C was primarily adapted to comply with national clinical and legal guidelines in relation to medical decision-making.

#### Section D my goals of future care

Similar to section C, section D ‘My goals of future care’ was also included in all 10 versions of the MPF as the topic was considered a key part of the AD. Section D consisted of two parts: a description scenario providing a context for the section and options. During the development phase, section D was modified on nine occasions (see Fig 1).

*Contextualising section D*. The first description scenario was inspired by the POLST form indicating *‘treatment preferences when I have a pulse and/or am breathing’* (Table 5, section D, version 1). In versions 3–5 (similar to version 6) the wording said *´When I have lost the capacity to make my own decisions and have a complication that I am unlikely to live through (survive)*, *I prefer the following measures to be taken´* (Table 5, section D, version 3–5). However, from version 7, a general statement on capacity covering sections C-F was included. It said: ´*If I were to become unable to communicate and express my preferences I would like the following issues to be taken into account*´ (Table 5, section D, version 7). Following this decision, the description scenario for section D focused on explaining the circumstances for which the options might be relevant, e.g. a potentially life-threatening complication (Table 5, section D, version 7–10). Throughout the process, the wording of capacity was much discussed and different words and phrases were proposed: ´capacity´, ´unconsciousness´, ´incompetence´, ´unable to make your own decisions´, ´unable to communicate´ etc. In this relation, a consortium member argued: *‘ACP may be useful even in cases in which the patient is not unconscious and to some extent still able to communicate*, *but he/she is not able to make autonomous choices due to internal factors (such as pain*, *medication*, *stress*, *disease progress) or external factors (e*.*g*., *pressure from family and friends) […]’*. Following this line of thought, the final wording applied was therefore to be ‘unable to communicate and express my preferences’.

*Restriction of options within section D*. One of the most significant modifications made in section D was the reduction from three to two options (Table 5, section D, versions 1 and 5). This change happened following continuous discussions within the consortium and with clinicians. It was concluded that the medical interventions presented within the ‘full treatment’ option were clinically irrelevant for the ACTION patient population and would rarely take place in the European settings. As explained by consortium members reporting clinician feedback: *‘In the UK [United Kingdom] it is very*, *very unlikely that a patient with this level of disease would ever end up in ICU [intensive care unit] […]’* and *‘about preferences for intubation or ventilation he [the physician] stated that […] they are rarely relevant for our target group […]´*. Moreover, these statements also lead to reflections within the consortium about the concept of ACP and ADs in relation to national practical implications in the ACTION countries. As asked by a consortium member: *‘[…] Do we want to know the patient´s wishes (no matter whether or not these can be followed) or do we want to only provide our patients with wishes which can potentially be followed*? *Allowing our patients to tick the third option*, *would in many ways feel like ‘deceiving’ and ‘cheating’ them […]*. *However*, *on the other hand it would clearly illustrate for the physician how far the patient is generally willing to go […]’*. Eventually, the option ‘full treatment to prolong my life’ was deleted from the MPF, as it was not seen as clinically relevant or plausible.

*Removal of specific locations for treatment/care in section D*. Another topic that was discussed in section D was that of linking the treatment options with locations (the hospital, at home etc.). Feedback from the Dutch facilitators and different clinicians indicated that the current options were too fixed. As a consortium member reporting clinician feedback recalled: *‘[…] The physician suggested to include the opportunity to express the preferences about comfort care to be provided in hospices*, *not only where the patient lives’*. However, providing a broader range of locations did not solve the problem of potentially making promises which might not be kept in the end. Especially for those patients with a strong preference for staying at home it was considered important to highlight the connection between more intensive treatment and the likelihood of hospitalisation. A consortium member therefore proposed: *‘[…] to leave out ‘places’ for section D and instead say something like ‘you need to be aware that the more treatment you wish to receive*, *the more likely it is that you will need to become hospitalized’*. Thus, to avoid limiting patients´ choices by providing “package deals” as well as providing promises that might not be kept, specific locations were replaced with phrases indicating that transfer to the hospital might take place (Table 5, section D, versions 1 and 10).

*Considering country-specific contexts in section D*. However, it was not only locations that were eventually left out, but also certain phrases or words within the options. One example is in relation to the topic of nutrition and fluids as stated within the Steering committee meeting minutes (Table 5, section D, version 1): *´taking in food and water by mouth´ is problematic in Italian context*. *Will be changed into*: *´taking in food and water´*. The quote referred to that before law no. 219/2017, artificial nutrition and hydration were not unanimously considered medical interventions but rather forms of basic care in Italy [13, 29]. This meant that the wording ´by mouth´ was not appropriate for the Italian situation, as it would exclude ANH within the comfort care option. As a standardized form across the ACTION countries, the MPF needed a wording acceptable for Italy. As a result, the wording was adapted in version 6. From version 7 and onwards, the phrase was nonetheless fully left out. Another example is that of the wording ´allow natural death´ included in version 3. This wording was deleted in version 8 as a consortium member asked: *‘Who is not in favour of natural death but does this automatically translate into a no to antibiotics against pneumonia*?*´*.

*From clinical to lay terminology within section D*. A final and far-reaching modification to section D that happened gradually over the developmental process was in relation to overall terminology. From applying clinical terminology to describe a set of specific treatment procedures in version 1, version 10 of the MPF uses lay terminology and refers to unspecified medical interventions determined appropriate by the physician (Table 5, section D, versions 1 and 10). This major change in language happened gradually based on the decisions and discussions already presented and the continuous critique from consortium members and clinicians who argued that the options were too complicated for clinical staff to explain and for patients to understand. Paradoxically, this change in language resulted in a consortium member reporting feedback from a clinician saying that the form was *‘not useful if written in lay language’*. Thus, a dilemma about how technical language should be remained.

As section D continued to be challenging, a pragmatic solution was eventually applied to help prevent erroneous use or interpretation of the patient´s goals and preferences; a space for free text was added below the options allowing the patient or facilitator to clarify when needed.

#### Section E my preferences regarding final place of care

The topics of ´place of care at the end of life´ and ´place of death´ were introduced in version 2. The sections were added based on discussions about how to best meet the needs of cancer patients, but were deleted in version 3, as it was decided that the same information could be documented in section F (´My other preferences´). In version 6, the topic ´preferred place of care´ was nonetheless reinstated as an independent section. In version 7, the wording ‘final’ was added (´preferred *final* place of care´ i.e. preferred place of death). Discussions about these adaptations could not be found within the material.

#### Section F my other preferences to be known by those who care for me

As part of the proposal for expanding the MPF to make it relevant to cancer patients, the topic of ‘other preferences’ was introduced in version 2. From version 3–7 of the form, instructions for what could be included within the section were listed. These instructions were modified several times (Table 5, section F, versions 3, 5 and 6), depending on the focus within the rest of the form and the needs expressed by consortium members. From version 8, no instructions were listed.

#### Sections that were deleted

Two sections, which originally were part of version 1 of the MPF, were deleted during the development process. The section ‘Artificially administered fluids and nutrition’ was deleted with the argument that there were already existing and clear procedures in place in the countries. The section ‘My Preferences as described above have been discussed with’ was deleted in version 9. The argument was that the patient could not have discussed the MPF with anyone, as the form was first presented to the patient during the ACTION RC ACP conversations.

## Discussion

Within an eight-month period from August 2014 to March 2015, the ACTION AD ’communication tool’, the MPF, was developed within the ACTION trial. Building on a fully data driven analysis, using qualitative methods, this article provides insight into the challenges and discussions faced as part of developing and adapting the ACTION AD specifically to a cancer population and, not at least, for use across six different European countries.

As described within the result section, several modifications took place during the development process. From the first to the final version of the MPF, only two revised themes remained (´cardiopulmonary resuscitation´ and ´life-prolonging treatment´). The development process ended up being more complex and time consuming than first expected. The result was a thematically comprehensive AD for cancer patients that was acceptable for all six ACTION countries and which mirrored the ACTION RC ACP conversations taking place within the ACTION trial.

Two arguments especially affected the development process. The first argument was the need for an AD that was thematically and clinically relevant for cancer patients, their PRs and clinicians; e.g. a disease specific rather than a generic AD. The second argument highlighted the need for making the US inspired form suitable for the clinical, legal and cultural frameworks in the six different European countries participating in the ACTION trial.

### The choice of a disease specific advance directive ’communication tool’

From our knowledge, the ACTION AD positions itself as one out of few disease specific ADs for cancer patients [5]. While far from widespread, discussions about or examples of disease specific ADs have continued to resurface over the years. Examples have been seen in relation to cancer, human immunodeficiency virus (HIV), dementia and end-stage renal disease (ESRD) [4–6, 30]. Common for these examples are descriptions of how generic ADs fail to capture relevant (disease specific) dimensions and decisions to be made and therefore risk being inadequate. These arguments are similar to the views expressed during the development process of the MPF, which as a result ended up being a more comprehensive form. Zwakman et al., who investigated how the MPF was completed by the ACTION patients, highlight exactly this characteristic and suggest this might be one of the reasons to why the ACTION patients completed the form in the first place [21]. Finding completion of an AD irrelevant or preliminary, together with lack of knowledge, have commonly been reported as barriers to AD completion [8, 31, 32]. Future research could ideally investigate not only patient acceptability and the effect of disease specific ADs [30], but also whether a disease specific AD could be an advantage in relation to some of the barriers previously mentioned.

### From a US POLST inspired framework to a European advance directive ’communication tool’

While the ACTION consortium members spoke of the MPF as an AD ’communication tool’, a POLST form was, as also previously highlighted, applied as the main inspiration for the first version of the MPF. A POLST form is a patient-held medical order for patients who are seriously ill, frail or at the end of life [33]. As stated by Mayora et al., who adapted the Oregon POLST form for Brazil, it is important to understand the differences between a POLST form and an AD [34]. They differ from each other in their language, context and use [35]. The use of the POLST form as an inspiration source might therefore explain some of the challenges met during the development process of the MPF, for example, the significant change from clinical to lay language and the general discussions about the aim, ownership and overall context of the MPF. Furthermore, using the POLST form as the main inspiration source also meant applying a US inspired framework for a multinational European study. The consequence was that some of the options described in the first versions of the MPF simply did not match the clinical reality within the European countries. As an example, the prescribed actions within the ´full treatment´ option in section D (´my goals of future care´) were considered unlikely to take place in the ACTION countries and was therefore deleted.

In addition, challenges concerning the topic of decision-making capacity were repeatedly discussed by the ACTION consortium. Which options should (and could) be offered to patients? And ultimately, who decided: patient or physician? Within the MPF, the physicians´ liability was prioritised above the choice of the patients. This was done to match the clinical reality within the ACTION countries where a patient´s wish for certain potentially lifesaving medical procedures, for example CPR, will not be fulfilled if deemed futile by the physician. As reported by Rietjens et al., patients do not necessarily have the same authority to refuse or request treatment across countries [1]. Cultural differences in relation to, for example, decision-making should therefore be taken into account when transferring and trying to adopt concepts or practices such as ACP and ADs. Similar points have been emphasised by Horn, who has provided accounts of how patient autonomy is valued differently across countries as well as examples of how cultural context and the law impact on physicians´ use of ADs [36, 37]. Thus, while standardisation is a key concept within multinational trials, the need for local adaptations also needed to be accommodated.

### The ACTION consortium—a European multinational consortium

As indicated within the background section, the ACTION countries had different starting points and prerequisites in relation ACP and ADs. The idea of a standardized AD ’communication tool’ for cancer patients to be used across six different European countries was therefore ambitious. In hindsight, it might even be relevant to ask the following question: ´did the ACTION consortium members hold the same definitions and ideas of the concepts of ACP and ADs?´. While a somewhat controversial question, two Delphi-studies taking place around the same time, demonstrated that internationally there was a need for a consensus definition of at least ACP and recommendations for its use [1, 38]. While there is not a clear answer to whether the ACTION consortium members held different definitions of ACP and ADs, the descriptions within the result section provide hints about different conceptualizations at play. As an example, this was seen in relation to the different discussions and views about the content and aim of the form, which were being expanded thematically during the development process. Such different understandings can have challenged the development process even further as consensus building became part of the mode of operation within the consortium: it was not only about developing an AD ´communication tool´, but also about agreeing on what that exactly meant. This course of events is not surprising as limitations and challenges for putting ADs in a cross-cultural perspective have previously been stressed [8, 39, 40]. It has also been shown that, in practice, ADs tend to adapt to the local cultural context which they are placed within [8, 41]. Within the ACTION trial, the use of the MPF as an informal communication tool rather than a formal AD ended up being characteristic for the majority of the ACTION countries.

### Strengths and limitations

A possible limitation of this study is the risk that pieces of relevant textual data have not been obtained or that not all discussions within the ACTION consortium have been documented. This article nonetheless captures an extensive amount of correspondence during about a year within a multinational research consortium and provides a data driven analysis about the development of something as complex as an AD that should be relevant across six different countries. Such detailed insights have rarely been presented within the literature on the development and/or adaptation of ADs.

## Conclusion

As part of the ACTION trial, an AD specifically for cancer patients to be used within a multinational context (i.e., six European countries) was developed. The development process was extensive, with no less than 10 draft versions of the document, numerous adaptations and an overall change from clinical to lay terminology. The result was a comprehensive, thematically broad AD ’communication tool’ designed for cancer patients across six European countries. The themes within the final version of the form were ´thoughts about living well´, ´hopes for current medical plan of care´, ´preferences for resuscitation´, ´goals of future care´, ´preferences for final place of care´ and ´other preferences´. The main arguments put forward during the development process were to adapt the AD to the needs of cancer patients while also assuring compliance with clinical, legal guidelines and local practices in the six European ACTION countries. Difficulties in the conceptualisation of ACP and ADs emerged relating to several questions, for example: do we want to know a patient´s goals and preferences, regardless of whether these can be followed? In the end, the ACTION AD was characterized by its multifaceted (hybrid) character, encompassing both elements of an AD as well as of the ACP process within the ACTION trial.

## Supporting information

S1 File Description of the ACTION respecting choices ACP intervention. (DOCX)

S2 File My Preferences form (version 1). (DOCX)

S3 File My Preferences form (version 10). (DOCX)

## List of abbreviations

ACP: advance care planning
AD: advance directive
ANH: Artificial nutrition and hydration
CPR: cardiopulmonary resuscitation
MPF: My Preferences form
PR: Personal Representative
POA-HC: Power of Attorney for Healthcare Document
POLST: Physician Orders for Life-Sustaining Treatment
RC: Respecting Choices
