# Supplementary material for: Development of an advance directive ’communication tool’ relevant for patients with advanced cancer in six European countries: Experiences from the ACTION trial
Source: PLoS One. 2022 Jul 28;17(7):e0271919. doi: 10.1371/journal.pone.0271919 (PMC9333298; doi:10.1371/journal.pone.0271919)
Supplement: S2 File — (DOCX) [file pone.0271919.s002.docx]

**(appropriate logo’s)**

**MY PREFERENCES**

* MY INDICATED PREFERENCES ARE RELEVANT IF:

| **THIS FORM HAS BEEN COMPLETED BY:**  My last name:  My surname:  My date of birth: .. (day) - …. (month) – …. year My gender: Male / Female  Address/ tel. number/ (registration number of patient?) | |
| --- | --- |
| **MY HEALTH CARE AGENT IS:**  Last name and surname of my Health Care Agent:  Date of birth of my Health Care Agent: .. (day) - …. (month) – …. year Gender: Male / Female  Address/ tel. number | |
| Section  **A**  Check one box only | **Treatment preferences when I am not breathing and have no pulse**  ☐ Resuscitate ☐ **D**o **N**ot attempt or continue any **R**esuscitation **(DNR)** |
| Section  **B**  Check one box only | **Treatment preferences when I have a pulse and/or am breathing ***  ☐ **Comfort Care only:** I am treated with dignity, respect and kept clean, warm and dry. Food and fluids are offered by mouth, but not forced upon me. Attention is paid to hygiene. Medication, positioning, wound care, and other measures are used to relieve pain and suffering. Oxygen, suction and manual treatment of airway obstruction may be used as needed for comfort. These measures are to be used where I live. If comfort measures fail, contact my physician.  ☐ **Comfort care with limited additional interventions aimed at prolonging my life:** Includes comfort care as described above. May include cardiac monitoring and oral/IV medications. Transfer to hospital if indicated, but no endotracheal intubation or long term life support measures. I understand that this type of care usually does not involve admission at an intensive care unit.  ☐ F**ull treatment to prolong my life:** Includes comfort care as described above plus measures that may prolong my life, such as endotracheal intubation, advanced airway, and cardioversion/automatic defibrillation at the intensive care unit.  ***Other instructions:*** *_________________________________________________*  *_________________________________________________________________* |
| Section  **C**  Check one box only | **Artificially administered fluids and nutrition** when I have a pulse and/or am breathing *** Comfort measures are always provided**  ☐ No feeding tube/IV fluids  ☐ Defined trial period of feeding tube/IV fluids  ☐ Long term feeding tube/IV fluids  ***Other instructions:*** ______________________________________________. |
| Section  **D** | My preferences as described above have been discussed with:  ☐ My health care agent:  ☐ My medical doctor (name):  ☐ Trained facilitator (name):  ☐ Other (specify): |

| My signature (mandatory) | Time and date signed |
| --- | --- |
| Signature of my health care agent (preferrably) | Time and date signed |
| **(NO signature of medical doctor)** | |

To be discussed:

- Include (more) goals of care? If yes, please provide suggestions.
- Include an explanation of the meaning of a facilitator? If yes, please provide suggestions.
- Include list of things the patient can do to communicate the plan? If yes, please provide suggestions.
- Include local explanation of the meaning of this form and how it relates to local forms? Please provide suggestions.
